# Being lonely or using substances with friends? A cross-sectional study of Hungarian adolescents’ health risk behaviours

**DOI:** 10.1186/s12889-015-2474-y

**Published:** 2015-11-07

**Authors:** Szabolcs Varga, Bettina F. Piko

**Affiliations:** Institute of Behavioural Sciences, Semmelweis University, Nagyvarad sqr. 4, 1089 Budapest, Hungary; School of Ph. D. studies, Semmelweis University, Ulloi str. 26, 1085 Budapest, Hungary; Department of Behavioural Sciences, University of Szeged, Szentharomsag str. 5, 6722 Szeged, Hungary

**Keywords:** Adolescent, Smoking, Drinking, Social behavioural factors, Psychological attributes

## Abstract

**Background:**

Studying adolescents’ health risk behaviours is oddly significant in Central and Eastern European countries, where the prevalence of smoking and drinking among 14–18 year old students is significantly high. The goal of our study is to examine the role of social psychological and social behavioural variables in health risk behaviours among Hungarian adolescents.

**Methods:**

Our sample was comprised of three high schools of Debrecen (the second largest city of Hungary). In all, 501 students filled in the questionnaire from 22 classes (14–22 years old). Students aged above 18 years were excluded for the purpose of the study, giving a total sample size of 471 high school students. Descriptive statistics and binary logistic regression analyses were conducted.

**Results:**

According to our results (1) social behavioural factors (namely, smoking and alcohol use of the best friend and peer group) proved to be better predictors of adolescents’ health risk behaviours as compared to the included social psychological attributes (2); among the latter ones, loneliness and shyness were negatively related with both smoking and drinking, while competitiveness was a predictor of drinking prevalence among boys.

**Conclusions:**

The findings suggest that social behavioural factors, including smoking and drinking of friends, are oddly important predictors of Hungarian adolescents’ health risk behaviours. According to our results, health policy should pay more attention to peer norms related to smoking and drinking during school health promotion. Developing health protective social norms may be an indispensable component of effective health promotion in high schools.

## Background

Studying adolescents’ health risk behaviours, including alcohol use and smoking habits, is oddly significant in Central and Eastern European Countries. According to an Italian study from 2013, 60 % of smokers smoked the first cigarette at the age of 18 or earlier. It is more aggravating that 33.6 % of smokers started to smoke at the age of 16 or younger [1]. A Hungarian study also established that social psychological and social behavioural factors correlated with health risk behaviours in adolescence; moreover, these two factors also often covaried. Social behavioural factor proved to be a better determinant, but a number of personality and psychological factors were also related to smoking and drinking [2]. Therefore, we aim to include both social psychological and social behavioural factors in the present study.

Among social psychological factors, loneliness, shyness, competitiveness and need to belong can potentially be contributors to youth health risk behaviours, according to Hungarian data [2, 3]. The relationship between social psychological aspects of personality and health risk behaviours was highly established (by studies from the USA and Hong Kong) among both adults and adolescents [4, 5]. Although the correlation between competitive attitudes and alcohol use was only indirectly investigated in many researches, the prevalence of alcohol use was higher among adults with type-A personality [4]. In adolescence, type-A personality is correlated with higher risk of both alcohol use and smoking. In addition, intense competitiveness is one of the main characteristics of type-A personality [4]. Only a few studies focused directly on the correlation between competitiveness and health risk behaviours, but significant relationship with smoking was already described by a study from the USA [6]. According to a Canadian sample, competitive attitude is also related with higher level of risk taking behaviours and sensation seeking, which could increase the risk of smoking and drinking [7]. A Hungarian study also reported significantly higher level of health risk behaviours among adolescents with competitive attitudes [3].

Shyness and loneliness are also associated with smoking. According to the Norwegian TOPP study survey among 14–17-year-old students, adolescents’ shyness increases the probability of smoking [8]. Loneliness has the same effect on smoking according to a study from Northern California, USA [9]. In addition, loneliness is also a risk factor of adolescent alcohol use, stated by Brazilian studies [10, 11]. On the other hand, studies (from the United States) about the correlation between adolescent alcohol use and shyness show inconsistent results [12, 13]. Not surprisingly, a study from the USA stated that shyness may lead to behavioural inhibition, that is, less health risk behaviours [14]. This was also described by a review paper in the case of loneliness, which has a particular significance in adolescence [15]; while some studies reported a positive relationship between substance use and loneliness, other studies failed to find differences in health risk behaviour patterns of lonely and non lonely youth. Moreover, these two variables are not independent. Lack of friends may increase the feeling of loneliness, and shyness can block adolescents in making connections with their classmates [9].

The relationship between need to belong and health risk behaviours has not been studied yet among adolescents. Thus, there is only indirect proof that higher level of (unmet) need to belong can be a risk factor of smoking and alcohol use among this age group: lack of relationships and extrovert personality was found to be connected with higher level of need to belong, according to an adolescent sample from the United States [16]. Studies from the USA and The Netherlands stated that these interrelationships may increase the chance of health risk behaviours [17, 18].

Besides social psychological factors, social behavioural variables can also have an influence on adolescents’ health risk behaviours. Several studies (from the USA, Canada, Poland, Finland and Kenya) established that cigarette and alcohol use of the peer group increase the chance of health risk behaviours [19–23]. This is true not only in the case of the same behaviour, but cross-correlation can also be verified by a sample among US adolescents. That is, friends’ smoking increases the risk of alcohol use, and vice versa [24, 25].

The goal of this study is to examine the relationship between social psychological and social behavioural variables and health risk behaviours among Hungarian high school students. We presume that in parallel with the international results, both groups of factors included in our study may correlate with health risk behaviours in this sample of adolescents. According to previous results, we hypothesize that among social psychological variables need to belong and competitiveness would correlate positively with smoking and drinking, while in the case of shyness and loneliness we would like to provide further information to the controversial results. We also expect that alcohol use and smoking of the adolescents’ best friend and peer group would positively relate to their health risk behaviours.

## Methods

### Ethics statement

Our research involved data from high school students. The method of the study was approved by the Institutional Ethical Boards (Institutional Ethical Board of Ady Endre High School, Institutional Ethical Board of Diószegi Sámuel High School and Institutional Ethical Board of Euro Baptist High School) and principals of the participated institutes in December, 2012 and January, 2013. Participation was confidential and voluntary.

### Study design and participants

Our sample contains data from 14 to 22-year-old students representing three different types of secondary school trainings (technical college, vocational education and a grammar school track) from three high schools of Debrecen (Hungary’s second largest city). The schools and classes of the sample were selected randomly. Firstly, the questionnaire was tested in a high school class to explore if any questions are not unequivocal for the participants. Based on the feedbacks, the test was reviewed. The study involved a vocational school with relatively low expectations from the suburb region, a technical college and an’elite’ grammar school from the city centre. The questionnaires were completed during January, 2013. In each class graduated teachers distributed the questionnaires to the students after a brief explanation, which included the goal of the study, technical information about the questionnaire and that participation is confidential and voluntary. They completed the questionnaires during the class period. Out of 503 possible eligible students from 22 classes, 501 students completed the survey (99.6 % response rate); only two adolescents refused to participate in the study. Participants aged above 18 years were excluded for the purpose of the study, giving a total sample size of 471 high school students (ages 14–18; mean = 16.21 years, SD = 1.13 years; 33 % males).

Due to the random sampling method, girls are overrepresented and technical college students are slightly underrepresented in the sample. Students’ distribution by age and grade represents the rates of the Hungarian high school student population [26].

### Measures

Dichotomous variables were used to measure the prevalence of students’ *health risk behaviours* (‘Do you smoke?’, ‘Do you drink alcohol?’) with 1 = ‘Yes’ and 2 = ‘No’ response categories. We also added a scale to measure the frequency of binge drinking (‘How many times did you drink a large amount of alcohol last month?’), with the response categories from 1 = ‘Never’ to 6 = ‘More than 10 times’. Answers from ‘Twice’ to ‘More than 10 times’ were coded into 1 = ‘Yes’ and other responses were coded into 2 = ‘No’ categories.

We included the following *social behavioural variables* to study the relationship between smoking and drinking behaviours of students: their best friends’ and peer group’s health behaviours. We measured the best friend’s prevalence (‘Does your best friend smoke/drink alcohol?’) and peer group’s frequency of smoking and drinking (‘How many of your friends smoke/drink alcohol?’). Answers about best friend’s health behaviours were coded with 1 = ‘Yes’ and 2 = ‘No’ response categories. Peer group related scale had five response categories from ‘None of my friends’ to ‘All of my friends’ and were coded from 1 to 3 (1 = ‘None/Minority of friends’, 2 = ‘Half of friends’, 3 = ‘Majority/All of friends’).

We also used four different *social psychological variables* that tapped into the desire/need to belong, shyness, competitiveness and loneliness.

The Need to Belong Scale, which was created to measure “the desire for acceptance and belonging for use in an experiment investigating reactions to potential acceptance and rejection.” [p. 2.] was previously used with the Chronbach’s Alpha values of 0.50–0.93 [16]. The scale contains 10 statements (e.g., ‘I try hard not to do things that will make other people avoid or reject me’ and ‘I want other people to accept me’), including 4 reversed items (e.g., ‘If other people don't seem to accept me, I don't let it bother me’). Answers were coded from 1 = ‘Not at all’ to 5 = ‘Entirely agree’ based on the level of agreement (5 to 1 for reversed items).

Secondly, Revised Cheek and Buss Shyness Scale (RCBS), with original reliability of 0.74–0.91 [27] was used to measure the adolescents’ level of shyness. The scale contained 13 items (e.g., ‘it is hard for me to act natural when I am meeting new people’), including 4 reversed ones. Answers were coded from 1 to 5 (5 to 1 for reversed items), with the same response categories.

The 14-item Competitiveness Scale was used with the same response categories and coding. The scale was used on adolescent samples with reliability coefficients of 0.90–0.93 [28, 29]. The scale’s reliability coefficient was 0.84, with the mean score of 44 (SD = 10.1) in the sample.

We also measured loneliness using the revised form of UCLA Loneliness Scale (previously used with reliability coefficient of 0.96) [30], which contains 20 statements (e.g., ‘I have nobody to talk to’), including 10 reversed items (e.g., ‘I can find companionship when I want it’). The answers were coded from 1 = ‘Not at all’ to 4 = ‘Entirely agree’. The scale yielded the mean score of 32 (SD = 8) and was reliable with Cronbach’s alpha coefficient of 0.85.

SPSS for MS Windows Release 16.0 software was used to conduct statistical analysis, including descriptive statistics and binary logistic regression analyses with a significance level of 5 %. The results of the binary logistic regression analyses are presented as a series of odds, with a baseline set to 1.0. A value < 1.0 shows that there is an inverse association between the factors of interest to the baseline odds while an odds ratio > 1.0 indicates positive association. 95 % confidence intervals were also calculated for significant relationships, depending on the criterion that the intervals do not include the odds = 1.0.

## Results

Table 1 presents the socio-demographic, health risk behaviour, social behavioural and social psychological characteristics for the secondary school student sample. Cronbach’s alpha values of the used social psychological variables in our sample were also included.

**Table 1:** General characteristics of Hungarian high school students (*n* = 471)

Characteristics	%	Mean	SD	n (response rate)	Chronbach’s Alpha
*Socio-demographics*					
Gender				470 (99.8 %)	
Boy	32.9 %				
Girl	66.9 %				
Age (14-18)		16.21	1.13	471 (100 %)	
Type of school				471 (100 %)	
Grammar	51.2 %				
Modern	42.9 %				
Technical	5.9 %				
*Health risk behaviours*					
Smoking				464 (98.5 %)	
No	64.7 %				
Yes	33.1 %				
Alcohol use				463 (98.3 %)	
No	30.1 %				
Yes	67.3 %				
*Social psychological variables*					
Need to belong (10-50)		34	5.7	440 (93.4 %)	0.60
Loneliness (20-80)		32	8	402 (85.4 %)	0.85
Shyness (13-65)		29.5	8.3	395 (83.9 %)	0.78
Competitiveness (14-70)		44	10.1	411 (87.3 %)	0.84
*Social behavioural variables*					
Best friend’s smoking				456 (96.8 %)	
No	51.4 %				
Yes	45.4 %				
Best friend’s alcohol use				450 (95.5 %)	
No	24.2 %				
Yes	71.3 %				
Friends’ smoking				442 (93.8 %)	
None/Minority of friends	49.5 %				
Half of friends	20.6 %				
Majority/All of friends	23.8 %				
Friends’ alcohol use				452 (96 %)	
None/Minority of friends	64.1 %				
Half of friends	11.9 %				
Majority/All of friends	20 %				

The sample contains data from 14 to 18-year-old students (mean = 16.21, SD = 1.13; female = 66.9 %, male = 33.0 %) representing three types of high school trainings (grammar = 51.2 %, modern = 42.9 %, technical = 5.9 %). 33.1 % of the sample smokes cigarette and 67.3 % drinks alcohol. These rates are in parallel with results of previous studies from the Hungarian high school student population. According to the ESPAD study from 2011, 37 % of Hungarian adolescents smoked and 61 % drank alcohol during the past 30 days [31]. Circa half (45.4 %) of students considered that their best friend smokes. This ratio is 71.3 % in the case of best friend’s alcohol use. Majority of adolescents considered that only minority or none of their friends smoked or drank alcohol. The response rates were high in the case of questions related to smoking and alcohol use (93.8 %–98.5 %).

Beyond descriptive statistics, binary logistic regression analyses were applied to study associations between the variables. Interrelationships between social behavioural and social psychological factors and health behaviours are presented in Table 2. Statistically significant relationships were found between both examined health risk behaviours (smoking, drinking and binge drinking) and social variables. The rates of smokers, drinkers and binge drinkers were significantly higher among those whose best friend smoked or drank alcohol, as compared to adolescents with non-smoking or non-drinking best friends. Peer group’s smoking is in a gradient-like relationship with all of the examined health risk behaviours. Higher rate of smoking friends is significantly related with higher odds of smoking, drinking and binge drinking. Gradient-like relationship was also found between friends’ drinking and adolescents’ alcohol use. If the majority or all of friends drink alcohol, the odds of smoking (*p* < 0.05; OR = 1.84; 95%CI = 1.14–2.97) and binge drinking (*p* < 0.001; OR = 2.87; 95%CI = 1.75–4.70) was also higher compared to other adolescents. According to these results there is a strong and positive association between smoking and drinking of friends (including the best friend and peer group) and the students’ health risk behaviours.

**Table 2:** Logistic regression estimates (OR) of predictors of health risk behaviours [OR (95 % CI), *n* = 471]

Predictor variables	Smoking	Drinking	Binge drinking
Best friend is smoking	16.7 (9.79-28.5)***	3.1 (2.02-4.74)***	2.08 (1.4-3.08)***
Best friend drinks alcohol	5.28 (2.85-9.8)***	24.2 (13.9-42.2)***	7.51 (3.75-15)***
Friends’ smoking			
None/Minority of friends	1	1	1
Half of friends	4.01 (2.32-6.95)***	2.11 (1.24-3.59)**	2.67 (1.61-4.44)***
Majority/All of friends	13.7 (7.93-23.6)***	3.86 (2.17-6.91)***	2.93 (1.81-4.74)***
Friends’ alcohol use			
None/Minority of friends	1	1	1
Half of friends	1.73 (0.96-3.11)	2.93 (1.42-6.02)**	1.7 (0.95-3.05)
Majority/All of friends	1.84 (1.14-2.97)*	4.35 (2.27-8.31)***	2.87 (1.75-4.70)***
Loneliness (cont.)	0.95 (0.92-0.98)**	0.95 (0.93-0.98)**	0.98 (0.95-1.02)
Shyness (cont.)	0.97 (0.95-0.99)*	0.97 (0.95-0.99)*	0.97 (0.95-0.99)*
Need to belong (cont.)	0.98 (0.94-1.01)	1 (0.96-1.03)	1 (0.93-1.03)
Competitiveness (cont.)	1 (0.99-1.03)	1.01 (0.99-1.04)	1.01 (0.98-1.02)
Gender			
Girl^a^	1	1	1
Boy	1 (0.67-1.49)	1.99 (1.29-3.07)**	1.57 (1.04-2.37)
Age (cont.)	1.24 (1.04-1.48)*	1.45 (1.21-1.74)***	1.12 (0.94-1.33)

^a^Reference category

*p < 0.05

**p < 0.01

***p < 0.001

Among the social psychological variables, shyness is significantly related to all examined health risk behaviours. Higher level of shyness is correlated with lower odds ratio of smoking (*p* < 0.05; OR = 0.97; 95%CI = 0.95-0.99), drinking (*p* < 0.05; OR = 0.97; 95%CI = 0.95–0.99) and binge drinking (*p* < 0.05; OR = 0.97; 95%CI = 0.95-0.99). There is also a significant relationship between loneliness and both smoking (*p* < 0.01; OR = 0.95; 95%CI = 0.92–0.98) and drinking (*p* < 0.01; OR = 0.97; 95%CI = 0.93-0.98). Both smoking (*p* < 0.05; OR = 1.24; 95%CI = 1.04-1.48) and drinking (*p* < 0.001; OR = 1.45; 95%CI = 1.21-1.74) correlate positively with age in our sample. Gender is related only to drinking; boys have a higher odds of drinking (*p* < 0.01; OR = 1.99; 95%CI = 1.29-3.07) than girls.

The sample was divided into two groups by gender, and binary logistic regression analyses were also conducted separately. Table 3 presents the regression estimates among boys. Comparing with the results of the whole sample, several differences were found. Social behavioural factors were still better predictors than social psychological variables, but the correlations between social behavioural variables and health risk behaviours are weaker. We have not found significant relationship between the best friend’s smoking and binge drinking, and between the best friend’s alcohol use and smoking. In addition, friends’ alcohol use does not correlate with smoking and drinking in this subgroup.

**Table 3:** Logistic regression estimates (OR) of predictors of health risk behaviours (boys) [OR (95 % CI), *n* = 155]

Predictor variables	Smoking	Drinking	Binge drinking
Best friend is smoking	11.7 (4.75-28.9)***	3.72 (1.54-9)**	1.78 (0.91-3.45)
Best friend drinks alcohol	2.86 (0.92-8.9)	45.8 (13.4-156)***	21.9 (2.82-170)**
Friends’ smoking			
None/Minority of friends	1	1	1
Half of friends	5.56 (1.96-15.8)**	4.07 (1.12-14.8)*	2.32 (0.95-5.66)
Majority/All of friends	18.4 (6.77-49.9)***	4.22 (1.34-13.3)*	2.62 (1.16-5.93)*
Friends’ alcohol use			
None/Minority of friends	1	1	1
Half of friends	2.58 (0.99-6.74)	1.15 (0.38-3.49)	1.07 (0.42-2.77)
Majority/All of friends	1.29 (0.57-2.92)	2.3 (0.8-6.62)	2.37 (1.06-5.32)*
Loneliness (cont.)	0.95 (0.91-0.99)*	0.93 (0.89-0.97)**	0.91 (0.86-0.96)**
Shyness (cont.)	0.98 (0.93-1.02)	0.95 (0.90-0.99)*	0.97 (0.93-1.01)
Need to belong (cont.)	0.99 (0.93-1.06)	1.06 (0.99-1.14)	1.05 (0.98-1.12)
Competitiveness (cont.)	0.99 (0.96-1.03)	1.06 (1.01-1.11)*	1 (0.96-1.04)
Age (cont.)	1.38 (0.97-1.95)	1.49 (1.02-2.16)*	1.67 (1.19-2.36)**

^a^Reference category

*p < 0.05

**p < 0.01

***p < 0.001

Among the social psychological variables, competitiveness has been found to be a risk factor of drinking. In addition, shyness does not correlate with smoking and there is a significant negative relationship between loneliness and binge drinking (*p* < 0.01; OR = 0.91; 95%CI = 0.86-0.96). Age also correlates with drinking (*p* < 0.01; OR = 1.49; 95%CI = 1.02-2.16) and binge drinking (*p* < 0.01; OR = 1.67; 95%CI = 1.19-2.36).

Odds ratios of logistic regression among girls are presented in Table 4. All of the examined social behavioural variables correlate significantly with all variables of health risk behaviours (that is, smoking, drinking and binge drinking) of the adolescent girls. On the other hand, less social psychological variables are related to health risk behaviours compared to boys. Significant relationship has been found only between loneliness and girls’ drinking (*p* < 0.05; OR = 0.96; 95%CI = 0.92-0.99), as well as shyness and prevalence of smoking (*p* < 0.05; OR = 0.97; 95%CI = 0.94-0.99). Age correlates only with drinking in this subgroup (*p* < 0.01; OR = 1.37; 95%CI = 1.11-1.68).

**Table 4:** Logistic regression estimates (OR) of predictors of health risk behaviours (girls) [OR (95 % CI), *n* = 315]

Predictor variables	Smoking	Drinking	Binge Drinking
Best friend is smoking	20 (10.3-38.9)***	2.91 (1.78-4.77)***	2.24 (1.36-3.68)**
Best friend drinks alcohol	6.69 (3.19-14)***	18.8 (10-35.1)***	5.5 (2.59-11.7)***
Friends’ smoking			
None/Minority of friends	1	1	1
Half of friends	3.53 (1.85-6.74)***	1.83 (1.01-3.34)*	2.92 (1.56-5.45)**
Majority/All of friends	11.9 (6.17-23.1)***	3.65 (1.85-7.19)***	3 (1.64-5.5)***
Friends’ alcohol use			
None/Minority of friends	1	1	1
Half of friends	1.33 (0.62-2.85)	4.63 (1.73-12.4)**	2.12 (1.01-4.84)*
Majority/All of friends	2.62 (1.23-4.16)**	5.36 (2.32-12.4)***	2.87 (1.52-5.43)**
Loneliness (cont.)	0.99 (0.95-1.02)	0.96 (0.92-0.99)*	0.97 (0.93-1)
Shyness (cont.)	0.97 (0.94-0.99)*	0.98 (0.95-1.01)	0.98 (0.95-1.01)
Need to belong (cont.)	0.97 (0.93-1.01)	0.99 (0.95-1.03)	0.98 (0.94-1.03)
Competitiveness (cont.)	1.02 (0.99-1.04)	1 (0.97-1.02)	1 (0.97-1.02)
Age (cont.)	1.21 (0.98-1.49)	1.37 (1.11-1.68)**	0.89 (0.72-1.11)

^a^Reference category

*p < 0.05

**p < 0.01

***p < 0.001

## Discussion

Our study focused on the correlations between social psychological and social behavioural variables and health risk behaviours in a sample of Hungarian adolescents. Among social behavioural variables, both smoking and drinking of the best friend and peer group increased the odds of health risk behaviours in both genders (the only exclusion is the peer group’s alcohol use among boys). Cross-correlations (alcohol use of adolescent – smoking of the best friend/peer group and vice versa) were also significant in both health risk behaviours among girls and alcohol use among boys. Binge drinking also correlated with social behavioural variables. Smoking and alcohol use of friends and the peer group increased the risk of binge drinking among both girls and boys. The same results were established in previous international and Hungarian studies [19–23, 32]. The role of these social factors proved to be contextual. Thus, in school classes where the prevalence of risk behaviours is higher, the society’s norms support or accept smoking and/or alcohol drinking. In these school classes social variables had a greater effect [33]. In comparison with other European countries, ratios of both alcohol use and smoking of Hungarian high school students are very high [31]. According to these results the norm of several Hungarian high school classes accepts or supports these behaviours.

Some of the included social psychological variables were also related to both adolescents’ alcohol use and smoking. Shyness among girls and loneliness among boys decreased the odds of smoking. Thus, our results are not consistent with some previous international studies [8, 9]. High level of feeling lonely decreased the risk of alcohol use among both genders. In addition, shyness also correlated negatively with this health risk behaviour among boys. Accordingly, the results of our study only partially support previous findings which described loneliness as a risk factor of alcohol use among adolescents [34–40]. But this result is in concordance with other studies that did not support this finding [15]. We should take into account that adolescence is a life period where peer norms and shared activities play an important role. While loneliness may play a negative role in adolescents’ psychosocial well being, it may prevent youth from common alcohol and cigarette use. Shyness may operate in a similar way [14]. On the contrary, adolescents’ competitive attitudes were linked with higher odds of alcohol use as well similar to previous studies on the link between competitiveness/hostility/Type A behaviour and health risk behaviours [4].

## Conclusions

Overall, the results of our study suggest the following: (1) social behavioural variables, including the best friend’s and peer group’s behaviours may strongly determine alcohol and cigarette use of Hungarian adolescents; (2) among social psychological variables high level of competitiveness may raise, while feeling of loneliness and shyness decrease the odds of risk behaviours.

The negative correlation between loneliness and shyness, and health risk behaviours may sound unexpectedly; however, this may be closely connected to the peer group effect and the role of the social network in an adolescent’s life. Firstly, our results, in parallel with several previous findings, established that the peer group may strongly determine adolescents’ health behaviours [19–23, 32]. In addition, based on the international statistics on high school students’ smoking and alcohol use, acceptance or support of these risk behaviours is significantly higher in Hungarian high school classes, compared to West European countries [31]. Consequently, there is a strong social pressure on Hungarian youth to smoke and drink. The strength of this social pressure on an individual student depends on the student’s role in the social network and the social hierarchy of the high school class [41–44]. Due to an adolescent’s shyness he/she may become marginalised in the class [42–45]. On the other hand, social pressure on this student is lighter, which may decrease the odds of health risk behaviours in the class where alcohol use and smoking is accepted or supported by peers [41]. This can be an explanation for the phenomenon that according to our results, health risk behaviours were negatively related to loneliness and shyness.

Several limitations of our study should be noted. Self-reported data was used on adolescents’ mental health and health behaviours, without any clinical diagnoses or objective source. In addition, our study is cross-sectional; therefore, we cannot provide cause-and-effect relationships. We think, for a better understanding of cause-and-effect relationships, more extensive studies should be conducted, using a longitudinal design. Among social psychological variables, the need to belong scale has a Cronbach’s alpha coefficient of 0.60 in our data. Thus, we think that this scale, which have been used with higher reliability in several international studies [16], needs further validation in Hungarian adolescent populations. The dichotomous nature of many of our variables makes it difficult to compare our results with studies using non-dichotomous variables. Thus, other variables should also be considered, such as frequency of smoking and drinking, socioeconomic status, lifestyle, social support or introversion-extroversion. Finally, our findings may have limited generalisability because of the study’s specific cultural context, sample size, and imbalance of the sample (e.g., grammar school students were overrepresented comparative of other types of high school student population of Hungary). On the other hand, we emphasize the relevance of population based studies from different countries, regions and cultures that may show different results in the pattern and strength of these correlations. All in all, these findings may increase our understanding of the role of social psychological and social behavioural factors in adolescents’ health risk behaviours. Finally, our results also suggest that there is a need for more research focusing on the social behavioural and social psychological context of adolescent health risk behaviours in population based studies. Particularly, using different non-western societies to map features of these associations.

With our study we tried to give a useful addition to the practice of high school prevention. According to our results, we suggest that teachers and actors of prevention policy should pay more attention to adolescents’ social psychological attributes and social (peer) norms of high school classes. These indicators correlate with the odds of health risk behaviours, especially alcohol use and smoking, which can have a negative effect on youth future physical health status [46]. In addition, our results also suggest that prevention cannot function effectively without actively involving the whole high school class, particularly where an adolescent’s peer group accepts or supports smoking and drinking. Thus, individual prevention cannot be effective without changing peer group norms into health protective ones.
